# Supplementary material for: Complexome profiling of the Chlamydomonas psb28 mutant reveals TEF5 as an early PSII assembly factor
Source: Plant Cell. 2025 Mar 17;37(6):koaf055. doi: 10.1093/plcell/koaf055 (PMC12164590; doi:10.1093/plcell/koaf055)
Supplement: koaf055_Supplementary_Data [file koaf055_supplementary_data.zip › psb28_interactive.zip!/plots/intensity/qualityPlots/peptides/_Cre05.g247950.t1.2.html]

### Cre05.g247950.t1.2

Trivial name:   
SeqCoverage [median]: 44.40  
Mass: 21.74 kDa  
  
 

| Sequence | MissedCleavage | Mass [Da] | wtIntensity | muIntensity | Score |
| --- | --- | --- | --- | --- | --- |
| HGDDGRPVSYR | 1 | 1257.59 | 1244.83 | 0.00 | 56.36 |
| IDFSAISGR | 0 | 964.50 | 9942.44 | 22148.02 | 114.89 |
| KSAGGSSSSSSSPAPK | 1 | 1420.68 | 792.31 | 1927.50 | 104.78 |
| MADDMAQVPYVTNPATHSMREPK | 1 | 2588.18 | 0.00 | 909.86 | 50.85 |
| TQIQQQQLQAK | 0 | 1312.71 | 3701.76 | 5650.30 | 182.92 |
| YVASTSPPSSAGAK | 0 | 1321.65 | 0.00 | 687.01 | 95.57 |
| YVSISDPK | 0 | 907.47 | 2503.43 | 6309.85 | 125.25 |
|  |  |  | 18184.8 | 37632.5 |  |
